# Synthesis and characterisation of a new benzamide-containing nitrobenzoxadiazole as a GSTP1-1 inhibitor endowed with high stability to metabolic hydrolysis

**DOI:** 10.1080/14756366.2019.1617287

**Published:** 2019-06-06

**Authors:** Veronica Di Paolo, Chiara Fulci, Dante Rotili, Francesca Sciarretta, Alessia Lucidi, Blasco Morozzo della Rocca, Anastasia De Luca, Antonio Rosato, Luigi Quintieri, Anna Maria Caccuri

**Affiliations:** a Department of Pharmaceutical and Pharmacological Sciences, University of Padova, Padova, Italy;; b Department of Experimental Medicine, “Tor Vergata” University of Rome, Rome, Italy;; c Department of Drug Chemistry and Technologies, “Sapienza” University of Rome, Rome, Italy;; d Department of Biology, “Tor Vergata” University of Rome, Rome, Italy;; e Department of Surgery, Oncology and Gastroenterology, University of Padova, Padova, Italy;; f Istituto Oncologico Veneto IRCCS, Padova, Italy;; g The NAST Centre for Nanoscience and Nanotechnology and Innovative Instrumentation, “Tor Vergata” University of Rome, Rome, Italy

**Keywords:** Nitrobenzoxadiazoles, GSTP1-1, TRAF2, glutathione, A375 human melanoma

## Abstract

The antitumor agent 6-((7-nitrobenzo[*c*][1,2,5]oxadiazol-4-yl)thio)hexan-1-ol (**1**) is a potent inhibitor of GSTP1-1, a glutathione *S*-transferase capable of inhibiting apoptosis by binding to JNK1 and TRAF2. We recently demonstrated that, unlike its parent compound, the benzoyl ester of **1** (compound **3**) exhibits negligible reactivity towards GSH, and has a different mode of interaction with GSTP1-1. Unfortunately, **3** is susceptible to rapid metabolic hydrolysis. In an effort to improve the metabolic stability of **3**, its ester group has been replaced by an amide, leading to *N*-(6-((7-nitrobenzo[*c*][1,2,5]oxadiazol-4-yl)thio)hexyl)benzamide (**4**). Unlike **3**, compound **4** was stable to human liver microsomal carboxylesterases, but retained the ability to disrupt the interaction between GSTP1-1 and TRAF2 regardless of GSH levels. Moreover, **4** exhibited both a higher stability in the presence of GSH and a greater cytotoxicity towards cultured A375 melanoma cells, in comparison with **1** and its analog **2**. These findings suggest that **4** deserves further preclinical testing.

## Introduction

We previously synthesised the nitrobenzoxadiazole (NBD) derivative 6-((7-nitrobenzo[*c*][1,2,5]oxadiazol-4-yl)thio)hexan-1-ol (NBDHEX, **1**), which proved to be a potent inhibitor of the human glutathione *S*-transferase (GST; EC 2.5.1.18) isoform P1-1 (GSTP1-1)^1^. Members of the GST family are known to catalyse conjugation of electrophilic molecules, including some anticancer drugs, with the tripeptide glutathione (GSH). This gives rise to inactive GSH conjugates which are rapidly extruded from the cell by efflux pumps belonging to the superfamily of ATP-binding cassette (ABC) transporters^2^^,^^3^. In addition, the isoform GSTP1-1 is overexpressed in several human tumours, and inhibits apoptosis by sequestering both the mitogen-activated protein kinase c-Jun *N*-terminal kinase (JNK), and the scaffold protein TNFα-receptor-associated-factor 2 (TRAF2)^4^. Since most of the currently used anticancer drugs induce cancer cell death by triggering apoptosis, restoration of apoptotic signalling pathways by targeting GSTP1-1 may represent an attractive strategy for therapeutic intervention^5^.

We have shown that compound **1** efficiently binds to GSTP1-1 and prevents formation of hetero-complexes involving JNK and TRAF2, thus avoiding inhibition of the apoptotic cascade mediated by these proteins^6–9^. Indeed, treatment with compound **1** exhibits remarkable pro-apoptotic activity towards cultured tumour cells, as well as therapeutic activity in human tumour xenograft models^10^. In order to increase the water solubility of **1,** two oxygen atoms have been inserted in its alkyl side chain, leading to 2-(2-(2-((7-nitrobenzo[c][1,2,5]oxadiazol-4-yl)thio)ethoxy)ethoxy)ethanol (MC3181; compound **2**). *In vivo* trials, demonstrated that therapeutic doses of **2** can be safely and effectively administered by both oral and intravenous routes to mice bearing human melanoma xenografts^11^^,^^12^. However, compared to **1**, compound **2** is characterised by a greater spontaneous reactivity towards nucleophiles, including GSH (Di Paolo, unpublished data), a characteristic that could accelerate its cellular efflux by the export pumps of the multidrug-resistance protein (MRP) family^13^ and compromise its therapeutic effectiveness. On the other hand, GSH transporters are also present at the blood–brain barrier (BBB)^14^ and GSH derivatives are efficiently exploited for drug delivery to the central nervous system through recognition by GSH transporters on the luminal side of BBB^15^. Moreover, some GSH analogs have been shown to have potent neuroprotective effects^16^. Thus, the therapeutic potential of the high reactivity of some NBD derivatives towards GSH is not fully understood at the moment.

On the basis of the hypothesis that lowering the reactivity of **1** and **2** towards nucleophilic aromatic substitution would prolong their lifetime in the body, and enhance their delivery to the target protein (i.e. GSTP1-1), we are currently focussing our efforts on developing new NBD derivatives endowed with a stability towards nucleophilic attack by GSH higher than that of **1** and **2**.

In this context, we recently reported the preparation and characterisation of 6-((7-nitrobenzo[c][1,2,5]oxadiazol-4-yl)thio)hexyl benzoate (MC2753; compound **3**) that is the benzoic ester derivative of **1**^17^. Introduction of a bulky benzoyl moiety in the side chain of the NBD scaffold of **1** has led to both a remarkable decrease in reactivity towards GSH, and a change of the mode of interaction with the target protein GSTP1-1. In particular, unlike **1**, compound **3** did not require GSH to trigger dissociation of the TRAF2-GSTP1-1 complex. Moreover, the σ-complex formed by reaction of **3** with GSH in the active site of GSTP1-1 was found to be much more stable than that of **1**. This latter feature implies a very slow enzymatic conversion of compound **3** into glutathionyl-NBD (GS-NBD), and thus, conceivably, a more prolonged disruption of the catalytic and non-catalytic functions of the target protein. Despite its interesting features, compound **3** is not suitable as drug candidate because of its high susceptibility to metabolism by carboxylesterases (CES; see “Results”), a class of ubiquitously-expressed enzymes that catalyse the hydrolysis of ester, thioester, amide, and carbamate linkages in a wide variety of endo- and xenobiotics^18^.

In an attempt to improve the hydrolytic stability of compound **3**, its ester group was replaced with an amide function, leading to *N*-(6-((7-nitrobenzo[*c*][1,2,5]oxadiazol-4-yl)thio)hexyl)benzamide (MC4351; compound **4**). Here we report the synthesis and initial characterisation of this novel compound, with particular attention to its spontaneous reactivity towards GSH, interaction with GSTP1-1, stability to human liver microsomal CES, and cytotoxic activity towards cultured human melanoma cells.

## Materials and methods

### Chemicals

Unless otherwise specified, all chemicals used throughout this work were purchased from Sigma-Aldrich Srl (Milan, Italy). GS-NDB was synthesised as previously described^1^.

### Chemistry

^1^H-NMR spectra were recorded at 400 MHz on a Bruker AC 400 spectrometer (Bruker, Billerica, MA); reporting chemical shifts in d (ppm) units relative to the internal reference tetramethylsilane (Me_4_Si). All compounds were routinely checked by TLC and ^1^H-NMR. TLC was performed on aluminum-backed silica gel plates (Merck DC, Alufolien Kieselgel 60 F_254_, Kenilworth, NJ) with spots visualised by UV light. Yields of all reactions refer to the purified products. All chemicals were of the highest purity. Mass spectra were recorded on an API-TOF Mariner by Perspective Biosystem; samples were injected by an Harvard pump using a flow rate of 5–10 µL/min, infused in the Electrospray system. Elemental analyses were performed by a PE 2400 (Perkin-Elmer, Waltham, MA) analyser and have been used to determine purity of the described compounds, which is >95%. Analytical results are within ±0.40% of the theoretical values.

### Preparation of N-(6-mercaptohexyl)benzamide (6)

To a solution of commercial 6-amino-1-hexanethiol hydrochloride **5** (1 eq, 0.589 mmol, 100.0 mg) and *N*,*N*-di*iso*propylethylamine (2.5 eq, 1.47 mmol, 0.256 mL) in dry DCM (1.5 mL) was added dropwise at 0 °C under nitrogen atmosphere a solution of benzoyl chloride (1 eq, 0.589 mmol, 0.068 ml) in dry DCM (2 mL). After stirring at room temperature under nitrogen atmosphere for 3.5 h, the reaction was stopped and the solvent evaporated. The residue was suspended in water and the product extracted with ethyl acetate (4 × 10 mL). The organic phase was then washed in sequence with potassium hydrogen sulphate 0.1 N (2 × 3 mL), sodium hydrogen carbonate saturated solution (2 × 3 mL) and brine (1 × 2 mL), dried over sodium sulphate, filtered and evaporated under reduced pressure. Finally, the crude product was purified by silica gel column chromatography eluting with a mixture ethyl acetate/petroleum ether 1:3 to give **6** as a colourless and viscous oil. Yield: 63.2%. ^1^H-NMR (400 MHz, CDCl_3_) δ ppm: 1.27–1.30 (t, 1H, S*H*), 1.32–1.44 (m, 4H, CONH(CH_2_)_2_C*H_2_*C*H_2_*(CH_2_)_2_SH), 1.53–1.60 (m, 4H, CONHCH_2_C*H_2_*(CH_2_)_2_C*H_2_*CH_2_SH), 2.43–2.49 (m, 2H, CONH(CH_2_)_5_C*H_2_*SH), 3.37–3.42 (m, 2H, CONHC*H_2_*(CH_2_)_5_SH), 6.03 (br m, 1H, CON*H*), 7.36–7.45 (m, 3H, C*H* benzene ring), 7.67–7.71 (m, 2H, C*H* benzene ring). MS (ESI), *m*/*z*: 238 [*M*+*H*]^+^.

### Synthesis of N-(6-((7-nitrobenzo[c][1,2,5]oxadiazol-4-yl)thio)hexyl)benzamide (4)

Pyridine (3.5 eq, 1.267 mmol, 0.073 mL) and *N*-(6-mercaptohexyl)benzamide **6** (1 eq, 0.362 mmol, 86.0 mg) were added to a solution of commercially available 4-chloro-7-nitrobenzofurazane (1 eq, 0.362 mmol, 72.3 mg) in 3 ml of a mixture ethanol:water (1:1). After stirring for 7 h at room temperature under nitrogen atmosphere, the ethanol was partially evaporated under vacuum. The resulting suspension was filtered and washed with water providing a crude product that was purified by silica gel column chromatography eluting with a mixture ethyl acetate/petroleum ether 45:55 and finally triturated with diethyl ether to yield **4** as a pure yellow powder. Yield: 43%. ^1^H-NMR (400 MHz, CDCl_3_) δ ppm: 1.41–1.45 (m, 2H, CONH(CH_2_)_2_C*H_2_*(CH_2_)_3_S), 1.52–1.64 (m, 4H, CONHCH_2_C*H_2_*CH_2_C*H_2_*(CH_2_)_2_S), 1.77–1.84 (m, 2H, CONH(CH_2_)_4_C*H_2_*CH_2_S), 3.18–3.22 (t, 2H, CONH(CH_2_)_5_C*H_2_*S), 3.39–3.44 (m, 2H, CONHC*H_2_*(CH_2_)_5_S), 6.06 (br m, 1H, CON*H*), 7.06–7.08 (d, 1H, C*H* benzoxadiazole ring), 7.36–7.38 (m, 2H, C*H* benzene ring), 7.41–7.43 (m, 1H, C*H* benzene ring), 7.67–7.69 (d, 2H, C*H* benzene ring), 8.32–8.34 (d, 1H, C*H* benzoxadiazole rings). MS (ESI), *m*/*z*: 401 [*M*+*H*]^+^. Anal. (C_19_H_20_N_4_O_4_S) Calcd. (%): C, 56.99; H, 5.03; N, 13.99; S, 8.01. Found (%) C, 57.15; H, 5.05; N, 13.88; S, 7.96.

### Evaluation of solubility and extinction coefficient (ε) of compound 4

The compound was first dissolved in 100% DMSO and then subjected to scalar dilution in buffer A (0.1 M potassium phosphate, pH 7.4, containing 0.1 mM EDTA and 0.1% (v/v) Triton X-100). The maximum solubility of these solutions was evaluated by recording the absorbance maxima (∼430 nm) and using a molar extinction coefficient calculated at the same wavelength, using diluted standard solutions prepared in buffer A (concentration range: 4–40 µM). The extinction coefficient was obtained by linear regression analysis of a plot of the maximum absorbance vs. compound concentration.

### Expression and purification of GSTP1-1 and TRAF2

Human GSTP1-1 expression was performed using the E. coli strain Top 10. Single colonies of freshly plated bacteria were used to inoculate 5-mL overnight cultures. These cultures were diluted into LB medium containing 100 µg/mL ampicillin and 50 µg/mL streptomycin, grown at 37 °C to an *A*_600_ value of 0.5 and induced by the addition of 0.5 mM isopropyl-β-d-thiogalactopyranoside (IPTG). Cell were grown at 37 °C for 18 h, harvested by centrifugation for 15 min at 7000×*g* and resuspended in 10 mM potassium phosphate buffer, pH 7.0, containing 1 mM EDTA and 10 mM DTT.

Human TRAF2 was expressed in *E. coli* BL21 (DE3) cells, transformed with the His-tagged TRAF2 C-terminal domain construct. These cells were grown in LB medium containing 30 µg/mL kanamycin sulphate. Cells were grown at 37 °C until the *A*_600_ value was 0.8, then the expression of TRAF2 was induced by the addition of 1 mM IPTG. Thereafter, cells were grown for 18 h at 30 °C. Afterwards, cells were harvested by centrifugation and resuspended in 20 mM Tris–HCl, pH 8.0, containing 150 mM NaCl, 20 mM imidazole, 10% glycerol, 1 mM DTT and an EDTA-free inhibitor of proteases. GSTP1-1 and TRAF2 cell suspensions were sonicated and clarified by centrifugation at 20,000×*g* for 15 min, at 4 °C and the resulting supernatant was further centrifuged at 100,000×*g* for 50 min at 4 °C.

GSTP1-1 was purified by affinity chromatography on a resin with immobilised GSH^19^. TRAF2 was purified on a Ni-NTA column^9^.

The TRAF2 and GSTP1-1 purity was analysed by SDS-PAGE. The protein concentration was determined by measuring the absorbance at 280 nm and using an extinction coefficient of 17,780 and 25,460 M^−1^ cm^−1^ for TRAF2 and GSTP1-1 monomers, respectively. Proteins were stored at –80 °C.

### Kinetic analysis

The enzymatic activity of GSTP1-1 (20 nM subunits) was spectrophotometrically assayed at 340 nm (ε = 9,600 M^−1^ cm^−1^) and at 25 °C, by measuring the rate of 1-cloro-2,4-dinitrobenzene (CDNB) conjugation with GSH as a function of time^20^. The assay mixture contained 1 mM GSH and 1 mM CDNB in 1 mL of buffer B (0.1 M potassium phosphate buffer, pH 6.5 containing 0.1 mM EDTA). The inhibitory potency of the compounds was determined by recording the activity of GSTP1-1 in the presence of increasing concentrations of the selected NBD derivative (0.01–20 µM).

### Evaluation of the stability of compounds 1–4 in the presence of GSH

Each test compound (10 µM) was incubated in a mixture (final volume, 0.2 mL) containing 0.1 M potassium phosphate (pH 7.4) and 1 mM GSH; control incubations were performed in the absence of GSH (buffer-only incubations). The reactions were conducted at 37 °C for different time intervals, and terminated by adding 10 µL of 20% (p/v) perchloric acid and 100 µL of ice-cold acetonitrile. Time 0 samples were prepared by adding all the components of the mixture to ice-cold test tubes containing 10 µL of 20% (p/v) perchloric acid, and 100 µL of acetonitrile. Samples were then centrifuged at 20,000×*g* for 10 min (4 °C) to separate the precipitates of potassium perchlorate, and aliquots of the supernatants were analysed for the disappearance of the test compound by HPLC with visible absorbance detection, as described below.

### Evaluation of the stability of compounds 3 and 4 in human liver microsomes

Compound **3** or **4** (10 µM) was incubated in a medium (final volume, 0.2 mL) containing 0.1 M potassium phosphate (pH 7.4) and 0.05 mg of protein/mL of pooled, mixed-gender human liver microsomes (Xenotech, LLC, Lenexa, KS, USA; HLMs); control incubations were performed in the absence of HLMs. The reactions were conducted at 37 °C for different time intervals and terminated by adding 0.1 mL of ice-cold acetonitrile. Time 0 samples were prepared by adding all the components of the mixture to ice-cold test tubes containing 0.1 mL of acetonitrile. Samples were then centrifuged at 20,000×*g* for 10 min (4 °C), and aliquots of the supernatants were analysed for the disappearance of the test compound by HPLC with visible absorbance detection, as described below.

The role of human liver microsomal CES in hydrolysis of compound **3** was investigated by incubating the test compound (10 µM) at 37 °C for 0 and 10 min in 0.2 mL of 0.1 M potassium phosphate buffer (pH 7.4) containing 0.05 mg of protein/mL of pooled, mixed-gender HLMs (Xenotech LLC) and the nonselective CES inhibitor benzil (50 µM)^21^; the incubation protocol and sample preparation for HPLC analysis were the same as described above. Control incubations were carried out in the absence of benzil.

### HPLC analysis

Analysis were conducted using a Hewlett-Packard series 1100 HPLC system (Agilent Technologies, formerly Hewlett-Packard, Palo Alto, USA) equipped with a degasser, a quaternary pump, an autosampler, a column oven, a variable wavelength UV–visible detector, and a diode-array detector; data were collected and integrated using the Agilent ChemStation software. Chromatographic conditions were as follows: column, Agilent Zorbax Eclipse XDB-C18 (3.0 × 150 mM, 5 µm; Agilent Technologies); mobile phase, 10 mM ammonium bicarbonate, pH 6.8/acetonitrile (90:10 v/v; solvent A) and acetonitrile (solvent B); elution programme, isocratic elution with 100% solvent A for 2 min, linear gradient from 0 to 70% solvent B in 8 min, followed by an isocratic elution with 70% solvent B until the end of the chromatographic run; post-run time, 7 min; flow rate, 0.4 mL/min.; injection volume, 30 µL; column temperature, 28 °C; detection, absorbance at 433 nm. Under the above conditions, retention times of compounds **1**, **2**, **3**, **4** and GS-NBD were 13.2, 11.4, 20.9, 14.4, and 5.9 min, respectively.

### ELISA for protein–protein interaction analysis

The formation of the TRAF2-GSTP1-1 complex was studied as previously described^9^. Briefly, 200 µL of His-tagged TRAF2 C-terminal domain (0.005 µM in 20 mM Tris–HCl, pH 7.6, containing 150 mM NaCl and 10% glycerol) were added to each well of a 96-well His-Sorb plate (Qiagen, Hilden, Germany) and incubated overnight, at 4 °C, on a rocking platform. Afterwards, wells were washed with PBS and incubated for 30 min with GSTP1-1 (concentration range from 0.1 to 2 µM) in 10 mM potassium phosphate buffer, pH 7.0, containing 0.1 mM EDTA. Incubation with GSTP1-1 was also performed in the presence of **1** (20 µM), **3** (20 µM) or **4** (20 µM), both in the absence and presence of GSH (1 mM). At the end of incubation, the wells were washed with PBS and then filled with 200 µL of a mouse anti-GSTP1-1 antibody (Cell Signalling, Beverly, MA, USA; 1:1000 in TBS containing 0.1% Tween and 5% non-fat dry milk) for 2 h at room temperature. Subsequently, wells were washed with PBS and incubated with an antimouse IgG antibody (Cell Signalling; 1:1000 in TBS containing 0.1% Tween and 5% non-fat dry milk) for 45 min at room temperature. The immunocomplexes were detected by the addiction of 200 µL/well of the 1-Step-Turbo TMB substrate solution (Pierce, Rockford, IL, USA). The reaction was stopped after 10 min by the addition of 50 µL of 2M H_2_SO_4_, and the absorbance was measured at 450 nm. Data were analysed by fitting to Equation (1) where ν is the percentage of the saturated binding sites of TRAF2; [*P*]*t* and [*L*]*t* are the total concentrations of monomeric GSTP1-1 and TRAF2, respectively^22^.

(1) $$\nu =100\frac{{\left[P\right]}_{t}+{\left[L\right]}_{t}+K_{d}-\sqrt{{\left({\left[P\right]}_{t}+{\left[L\right]}_{t}+K_{d}\right)}^{2}-4{\left[P\right]}_{t}{\left[L\right]}_{t}}}{2{\left[L\right]}_{t}}$$

### Cytotoxicity experiments

The human melanoma A375 cell line was obtained from the American Type Culture Collection (ATCC, Manassas, VA, USA). Cells were cultured in Dulbecco’s Modified Eagle’s Medium (DMEM), supplemented with 10% foetal bovine serum FBS (v/v), 2 mM l-glutamine, 100 U/mL penicillin and 100 µg/mL streptomycin, at 37 °C in a 5% CO_2_ humidified atmosphere. For cell viability studies, A375 (2.0 × 10^4^ cells/cm^2^) were seeded in 96-well plates and, after 24 h at 37 °C, were exposed to different concentrations of NBD-derivatives (0.05–25.0 µM) for 48 h. After incubation, cell survival was evaluated by the sulforodamine B (SRB) assay^23^, as previously described^6^.

### Molecular docking

Molecular docking simulations were used to asses binding affinities and poses using Autodock Vina^24^, a recent and efficient implementation of docking software from Molecular Graphics Laboratory (MGL) of The Scripps Research Institute. Receptors were prepared starting from crystal structures of the apo-GSTP1-1, PDB code 5DCG^25^ and the GSH-NBDHEX complexed structure, PDB code 3GUS^26^. Water and cosolutes were removed from the structure which was further corrected for multiple occupancies using UCSF Chimera^27^.

**Figure 1.**
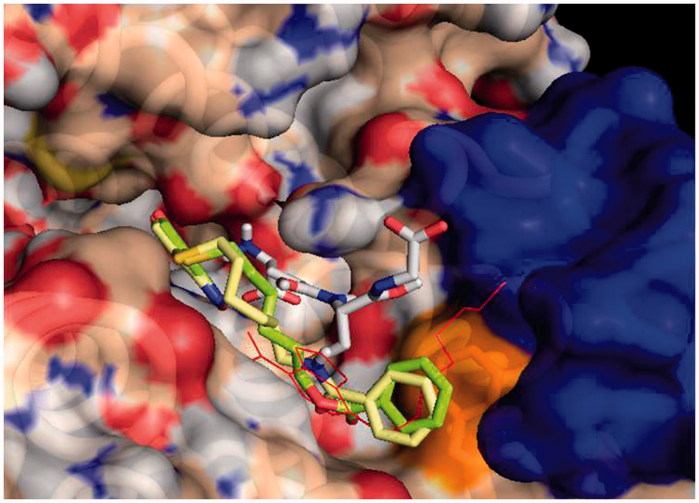
Protein-ligand docking analysis: best binding poses for **3** and **4**. Both compounds show a similar arrangement in their lowest energy poses, with a shifted NBD moiety and the benzoyl terminal making extensive contact with Phe 8. Crystal position for **1** is shown in red lines for reference, GST is shown in surface representation, with contributions coming from Phe 8 in orange (also shown in sticks) and those from Helix 2 in blue. GSH is also shown in sticks representation.

**Figure 2.**
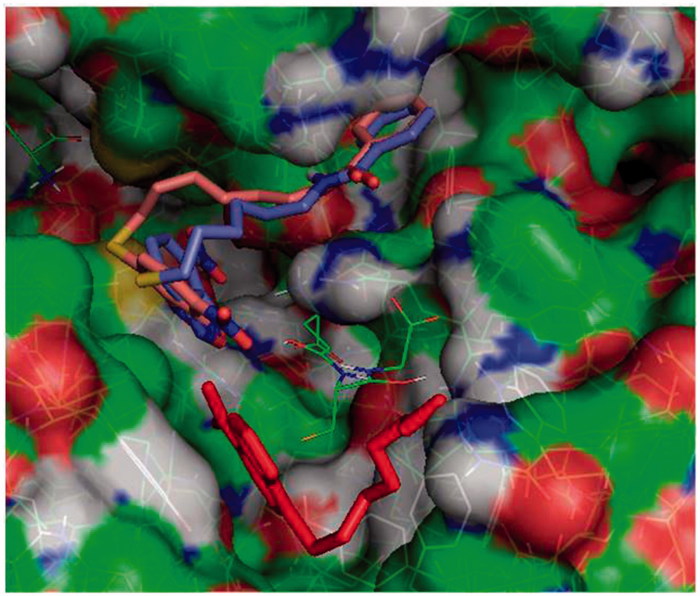
Protein-ligand docking analysis: alternative binding mode for **3** and **4**. Both **3** (brick) and **4** (purple) exhibit a higher energy alternative binding pose which stretches toward the other monomer of GST. Compound **1** crystal position is shown in red sticks for reference and GSH is indicated in lines.

The structures of **3** and **4** were initially built using the Chimaera programme and then processed in AutoDockTools assigning partial charges using the Gasteiger method. All suitable ligand dihedrals were allowed to rotate. The docking grid was centred on the GST active site and large volume (22.75 × 22.75 × 28.25 Å) was used to allow extensive conformational sampling. The number of binding modes for each run was set to 15 and exhaustiveness parameter to 25. To enhance sampling five runs were executed independently, each starting from a different random seed, and the results were merged. The final 75 structures were ranked according to the most favourable docking energy and clustered according to relative root-mean-square-deviations. Docked structures were analysed with AutoDockTools and Pymol which was also used to prepare Figures 1 and 2.

### Data analysis

Unless specified otherwise, experiments were repeated at least three times, and results are presented as mean ± SD. The Student's *t* test for unpaired data was used for comparison of the mean values between two groups. Differences were considered statistically significant when *P* < 0.05.

Metabolic stability of each test compound, expressed as percentage of compound remaining (mean ± SD; *n* = 3), was calculated by comparing the corresponding chromatographic peak area at each time point relative to that at time 0 min. Disappearance half-live values of the studied NBD derivatives were calculated by non-linear regression analysis of the data, using a one-phase exponential decay model^28^, provided by GraphPad Prism (GraphPad Software, San Diego, CA, USA).

## Results

### Chemistry

**Scheme 1.**
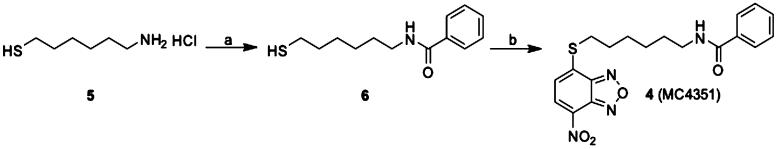
Preparation of compound **4**. (a) Benzoyl chloride, *N*,*N*-di*iso*propylethylamine, dry DCM, N_2_, 0 °C to rt; (b) 4-chloro-7-nitrobenzofurazane, pyridine, ethanol/water 1:1, N_2_, rt.

The benzamide derivative **4** has been prepared starting from the commercial 6-amino-1-hexanthiol **5**, that has been first acylated with benzoyl chloride to provide the intermediate *N*-(6-mercaptohexyl)benzamide **6**, that has been finally converted into the desired *N*-(6-((7-nitrobenzo[*c*][1,2,5]oxadiazol-4-yl)thio)hexyl)benzamide **4** through the nucleophilic displacement with the commercially available 4-chloro-7-nitrobenzofurazane (Scheme 1).

### Comparison of chemical and physical properties of compounds 1–4

**Figure 3.**
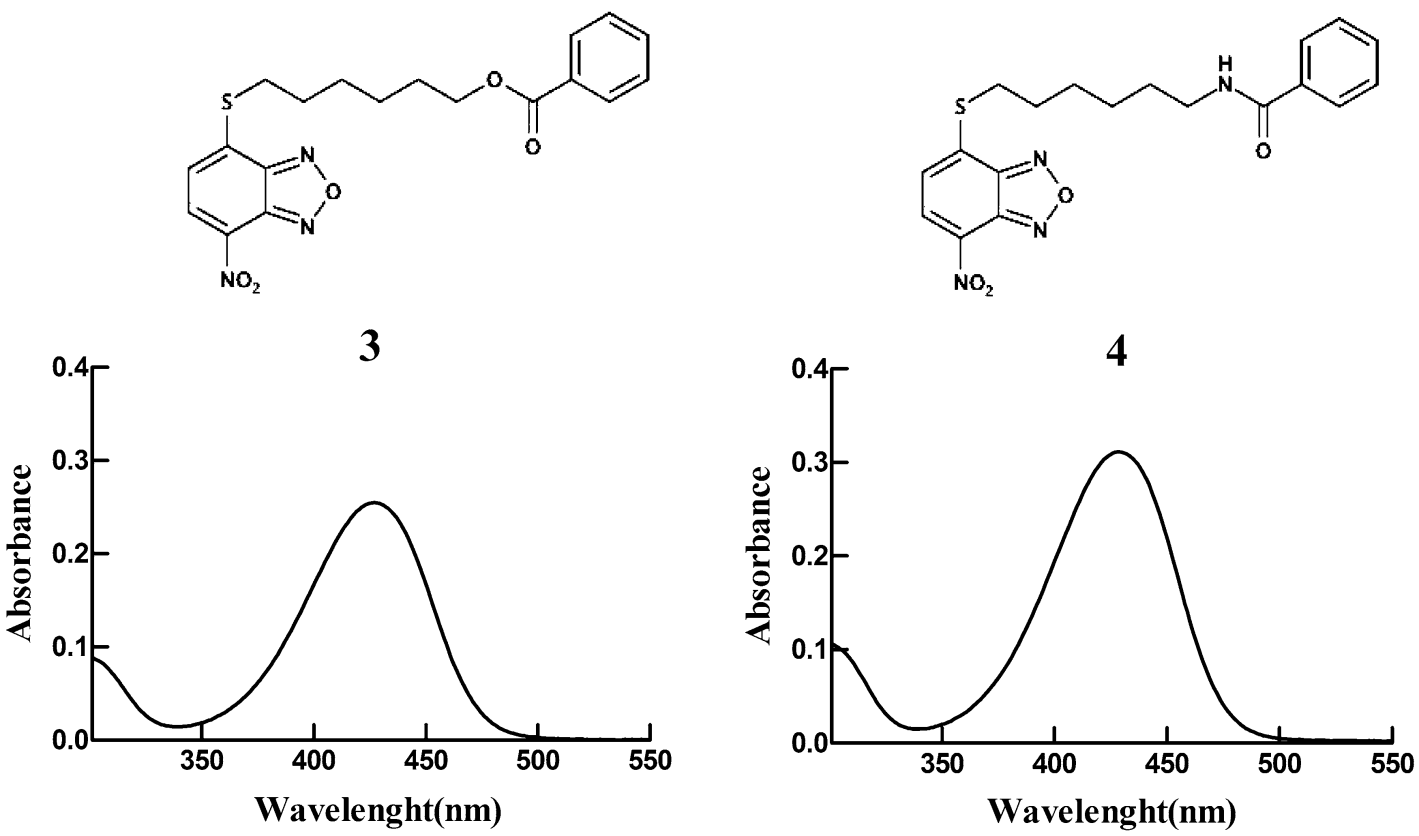
Spectrophotometric analysis. Structures and UV–visible spectra of 3 and 4 (20 µM), dissolved in buffer A, pH 7.4. Both spectra were recorded at 25 °C.

The molecular weights, extinction coefficients, and the aqueous solubility profiles of **4**, of its parent compound **1,** and its analogs **2** and **3** are reported in Table 1. The structures and UV–vis spectra of **3** and **4** are also shown in Figure 3. Compound **4** showed an extinction coefficient at 430 nm of 14.9 (mM^−1^ cm^−1^) and a solubility limit of approximately 0.08 mM, a value slightly higher than that of **3** (0.05 mM)^17^.

**Table 1.** Molecular weights, extinction coefficients and solubility limits.

Compound	Molecular weight (Da)	Extinction coefficient (ϵ) (mM^–1^ cm^–1^)	Solubility (mM)
**1** (NBDHEX)	297.3	15.0^a^	0.1^e^
**2** (MC3181)	329.3	16.4^b^	5.0^e^
**3** (MC2753)	401.4	12.5^c^	≤0.050^f^
**4** (MC4351)	400.5	14.9^d^	≤0.080^f^

^a^ϵ at 433 nm.

^b^ϵ at 425 nm reported by De Luca et al.^11^

^c^ϵ at 430 nm reported by Fulci et al.^17^

^d^ϵ at 430 nm.

^e^0.1 M K-Phosphate buffer, pH 7.4, reported by De Luca et al.^11^

^f^0.1 M K-Phosphate buffer, pH 7.4, containing 0.1% (v/v) Triton X-100.

### Compound 4 exhibits a lower reactivity towards GSH than compounds 1 and 2

**Figure 4.**
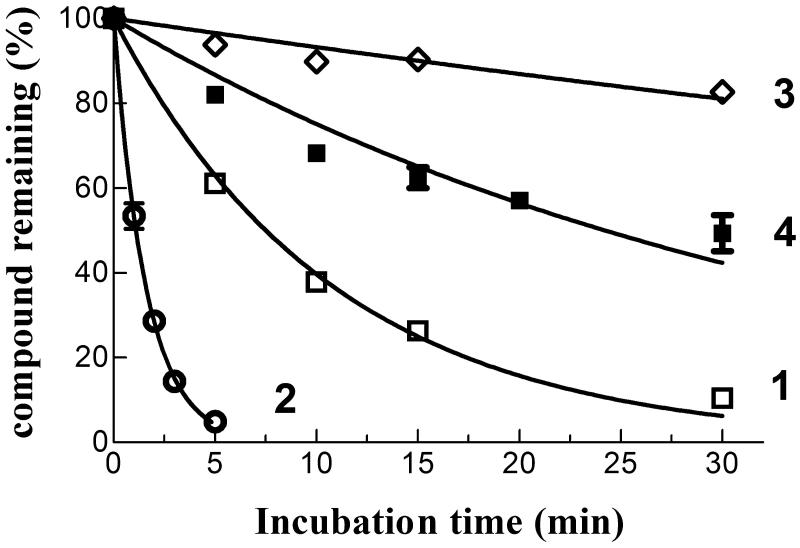
Spontaneous reactivity of compounds **1–4** towards GSH. Each NBD derivative (10 µM) was incubated with 1 mM GSH in 0.1 M potassium phosphate (pH 7.4) at 37 °C for different time intervals. The data are expressed as percentage of compound remaining at each time compared with time 0 min, and represent the mean ± SD of three independent determinations. Error bars smaller than the symbols are not visible.

The spontaneous reactivity of **4** towards GSH was compared with that of compounds **1**–**3**; in these HPLC-based trials, each NBD derivative (10 µM) was incubated at 37 °C for different time intervals in a phosphate buffer (pH 7.4) containing a physiological intracellular concentration of GSH (1 mM). Both compound **1** and **2** reacted quickly with GSH, with estimated half-lives of ∼7 and 1 min, respectively (Figure 4); loss of both compounds from the incubation mixture correlated with the appearance of a new prominent chromatographic peak having a retention time and an absorbance spectrum identical to that of authentic GS-NBD (not shown). Compound **4** was remarkably less reactive towards GSH than **1** and **2**, with an estimated half-life (∼30 min) closer to that of compound **3** (∼100 min). Thus, the isosteric replacement of the ester moiety of **3** with an amide function is accompanied by only a limited increase of spontaneous reactivity towards GSH under physiological conditions of pH, temperature and concentration of the tripeptide^3^.

### Compound 4 displays GSTP1-1 catalytic activity inhibition properties superior to those of compounds 2 and 3

**Figure 5.**
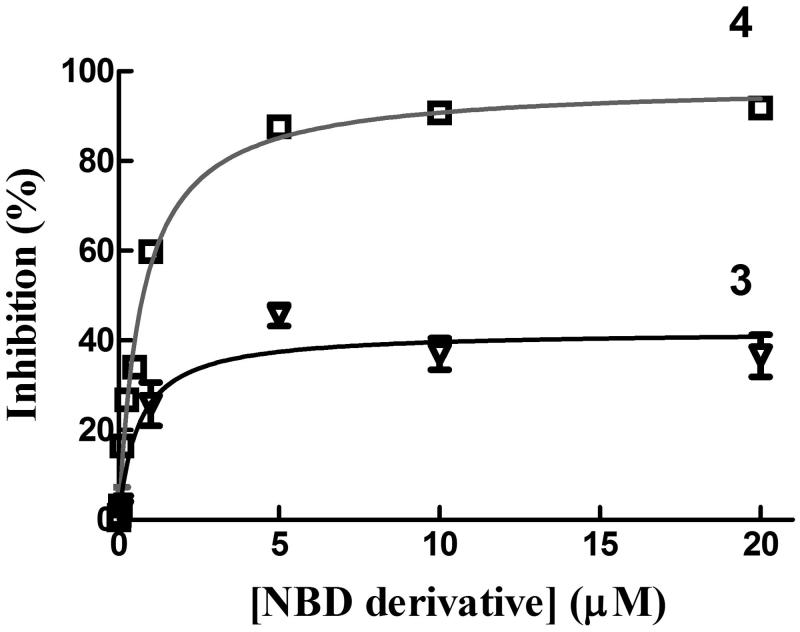
Inhibition of GTP1-1 conjugation activity. Inhibition of GSTP1-1 by 3 and 4 was evaluated at 25 °C. The assay mixture contained 1 mM GSH, and 1 mM CDNB in 1 mL of buffer B (0.1 M potassium phosphate buffer, pH 6.5, containing 0.1 mM EDTA). Data points represent the mean ± SD from three independent experiments. Error bars smaller than the symbols are not visible.

The chemical and physical properties of compounds **3** and **4** have been evaluated in a buffer containing 0.1% (v/v) Triton X-100. However, we previously found that the presence of a nonionic surfactant decreased the affinity of GSTP1-1 for both compounds **1** and **3**^17^. Therefore, GSTP1-1 activity inhibition experiments were performed by dissolving both compound **3** and **4** in a detergent-free buffer. Under these conditions, compound **3** inhibited GSTP1-1 conjugation activity no more than 50% (Figure 5), as previously observed^17^. In contrast, like compound **1** (*K_i_*^app^=0.8 ± 0.1 µM)^11^, compound **4** completely inhibited GSTP1-1 enzyme activity (*K_i_*^app^=0.7 ± 0.1 µM; Figure 5). Therefore, despite its structural similarity with **3**, compound **4** displays inhibitory properties towards GSTP1-1 catalytic activity comparable to those of its parent compound, i.e. compound **1**. Notably, the GSTP1-1 inhibition potency of compounds **1** and **4** was ∼3-fold higher than that of the more water-soluble analog **2** (*K_i_*^app^=2.6 ± 0.3 µM; Table 2)^11^.

**Table 2.** GSTP1-1 and cell growth inhibition data.

Compound	GSTP1-1 inhibition assay IC_50_ (µM)	A375 cytotoxicity assay IC_50_ (µM)
**1** (NBDHEX)	0.8 ± 0.1^a^	0.37 ± 0.02
**2** (MC3181)	2.6 ± 0.3^a^	0.77 ± 0.03
**3** (MC2753)	0.6 ± 0.3^b^	–
**4** (MC4351)	0.7 ± 0.1	0.24 ± 0.01

^a^IC_50_ values reported by De Luca et al.^11^

^b^IC_50_ value reported by Fulci et al.^17^

### Compound 4 disrupts the interaction between GSTP1-1 and TRAF2 both in the presence and in the absence of GSH

**Figure 6.**
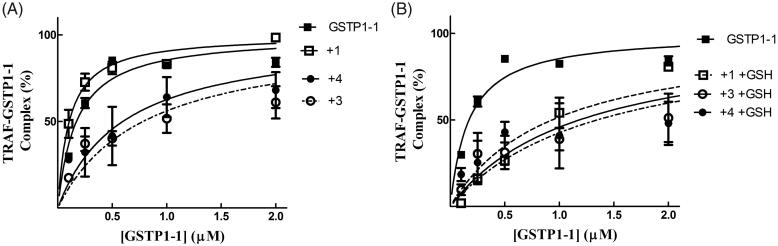
Inhibition of the TRAF2-GSTP1-1 interaction. (A) His-tagged TRAF2 (0.005 µM) was immobilised on Ni-NTA-coated plates and incubated at pH 7.0 with increasing amounts of GSTP1-1 (from 0.1 to 2 µM) in the absence (-■-) or in the presence of compounds **1** (-□- 20 µM), **3** (-^- 20 µM) or **4** (-●- 20 µM). (B) The same experiments were repeated also in the presence of GSH (1 mM). In this case, the binding trend of **1** overlaps those obtained with **3** and **4**. Data points represent the mean ± SD from three independent experiments. Error bars smaller than the symbols are not visible.

We have previously shown that compound **1** is capable of reducing the affinity of GSTP1-1 for TRAF2 only in the presence of GSH. By contrast, compound **3** was found to be very efficient in weakening the interaction between TRAF2 and GSTP1-1 even in the absence of the tripeptide^17^. Noteworthy, compound **4** showed a behaviour comparable to that of **3**, i.e. inhibited TRAF2-GST-P1-1 complex formation both in the absence and in the presence of GSH (Figure 6).

### Compound 4 displays a high stability in the presence of human liver microsomes

**Figure 7.**
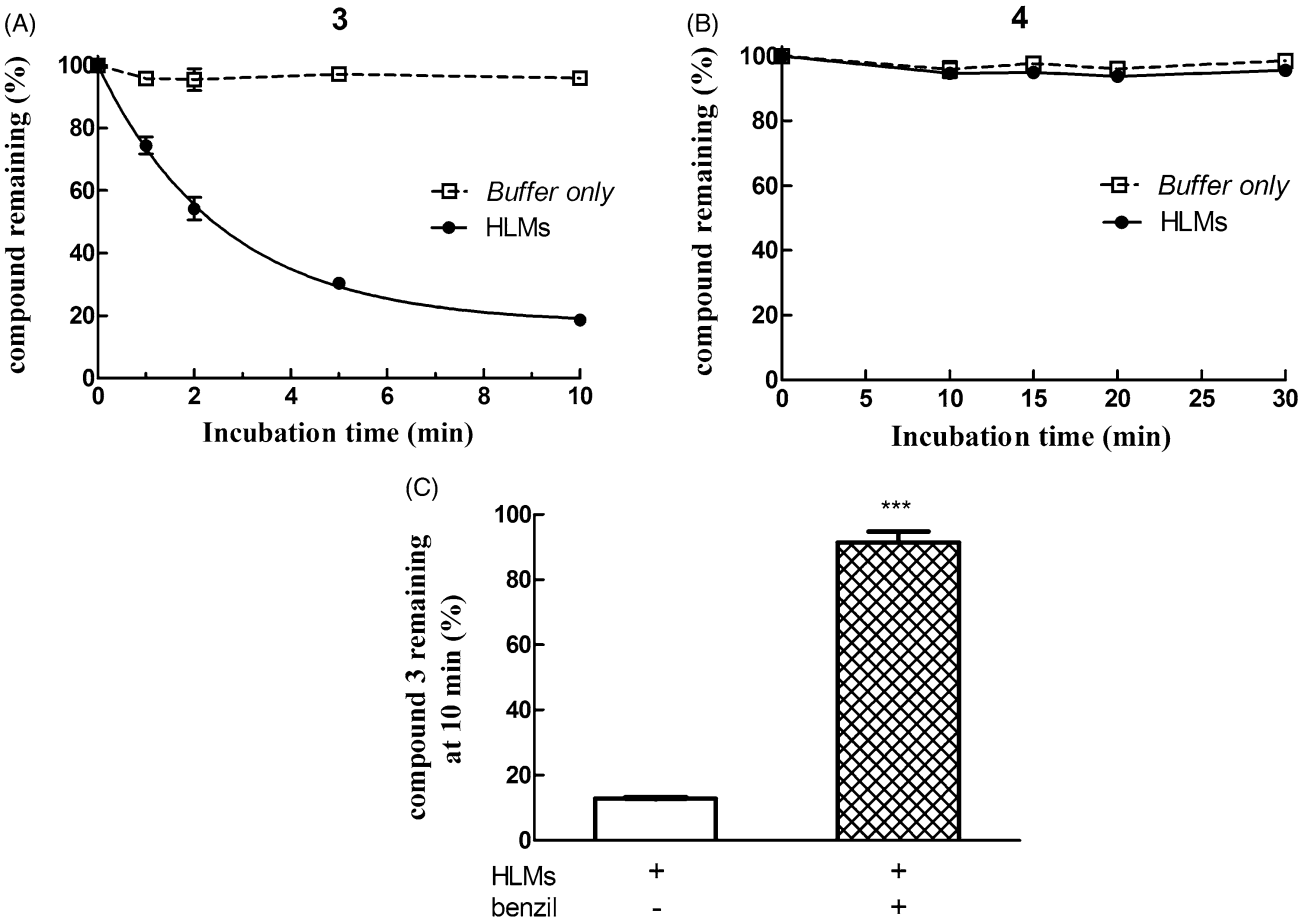
Assessment of metabolic stability of compounds **3** and **4** in human liver microsomes (HLMs). Compound **3** (panel A) or **4** (panel B) was incubated with pooled, mixed-gender HLMs (0.05 mg of protein/mL) in 0.1 M potassium phosphate buffer, pH 7.4 (-•-) or buffer only (-□-) at 37 °C for various time intervals (*n* = 5). The final concentration of each NBD derivative in the incubation mixture was 10 µM. To evaluate the role of CES on depletion of **3** in human liver microsomal incubations, the NBD derivative (10 µM) was incubated at 37 °C with pooled, mixed-gender HLMs (0.05 mg of protein/mL) in 0.1 M potassium phosphate buffer (pH 7.4) for 0 and 10 min, both in the absence and in the presence of 50 µM benzil (panel C). The data are expressed as percentage of compound remaining at each time compared with time 0 min, and represent the mean ± SD of three independent determinations. Error bars smaller than the symbols are not visible. ***P < 0.001 vs. control (minus benzil) at 10 min.

As shown in panel A of Figure 7, the ester linkage-containing NBD derivative compound **3** was stable when incubated at 37 °C in phosphate buffer (pH 7.4) only, but time-dependently disappeared when HLMs where included in the mixture, giving rise to compound **1** (not shown). Metabolic depletion of **3** was remarkably inhibited by 50 µM benzil^21^, a finding indicating the involvement of one or more human liver microsomal CES in the hydrolytic reaction (Figure 7, panel C). In contrast, compound **4** (i.e. the amide analog of **3**) was quite stable both in phosphate buffer only and in the presence of HLMs (Figure 7, panel B).

### Compound 4 exhibits remarkable antitumor activity towards cultured human melanoma cells

**Figure 8.**
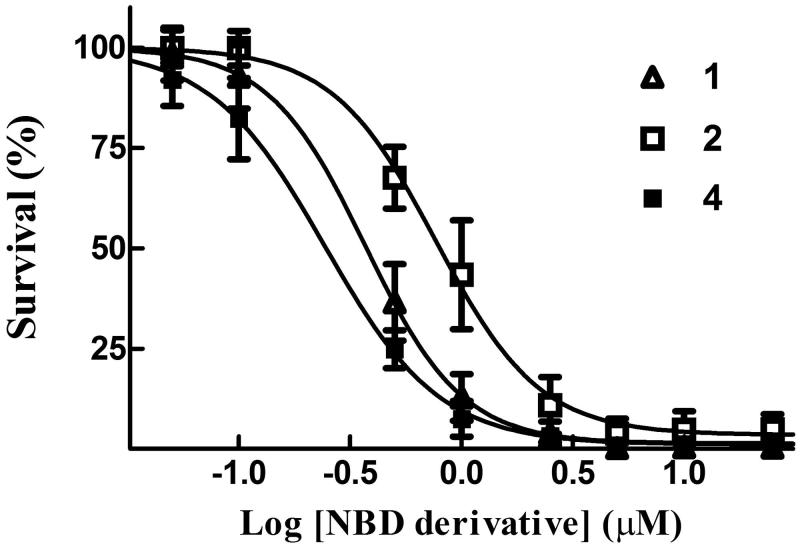
Cytotoxicity assay. Dose–response curves were obtained from human melanoma A375 cells treated with graded concentrations of compounds **1**, **2** and **4**. After 48 h of treatment, cell growth was evaluated by the SRB assay and expressed as a percentage of the control values. Data represent the mean ± SD of three independent determinations. The dose–response profiles allowed the calculation of the IC_50_ values reported in Table 2.

A last set of *in vitro* experiments compared the cytotoxicity of compound **4** with that of compounds **1** and **2** in A375 human melanoma cells, using an SRB assay; results of these trials are summarised in Figure 8 and Table 2. Interestingly, the IC_50_ value of **4** (0.24 ± 0.01 µM) was ∼3- and 2-fold lower than that of **2** (0.77 ± 0.03 µM) and **1** (0.37 ± 0.02 µM), respectively.

### Docking results

Molecular docking simulations were used to try to capture differences in binding modes that could account for different dynamical and functional behaviour of **3** and **4**. Results on the GSH loaded enzyme show that both inhibitors bind with high affinity in an elongated pose along the cleft of the H binding site of GSTP1-1. The NBD moiety is slightly deeper in the cavity with respect to that of the parent compound **1**, and the terminal benzoyl moiety is in close contact with the hydrophobic Phe 8 residue (Figure 1). The interaction energy reaches –8.4 and –8.8 kcal/mol for **3** and **4,** respectively. Consistently with our previous results, a different binding mode was also found for **3** and **4**, albeit at lower binding energies (with maximum at –7.4 and –7.8 kcal/mol, respectively). In this mode the NBD is displaced from the crystal position and the benzoyl moiety is stretched toward the cavity lying behind Lys 102 of the other monomer, towards Tyr 49 (Figure 2).

## Discussion

Over the last few years, many efforts have been made to improve the delivery of antitumor NBD derivatives through an increase of their solubility in aqueous vehicles. These endeavours resulted in compounds with a greater spontaneous reactivity towards nucleophilic attack by GSH, a characteristic that conceivably translates into a faster *in vivo* conversion to rapidly excreted GSH conjugates.

More recently we synthesised and characterised compound **3**, i.e. the benzoyl ester derivative of the prototypical antitumor NBD derivative **1**. Introduction of the bulky benzoyl moiety in the side chain of compound **1** led to a compound endowed with different features, including a remarkable stability towards GSH under physiological conditions^17^. Unfortunately, compound **3**, like several other carboxyl ester linkage-containing compounds, is highly susceptible to hydrolysis catalysed by CES^29^ (see panels A and C of Figure 7), a feature limiting its availability in biological systems. However, the isosteric replacement of the carboxyl ester linkage of **3** with an amide moiety was successful in improving the stability to metabolic hydrolysis. Indeed, compound **4**, the amide analog of **3**, was found to be quite stable to human liver microsomal esterases (panel B, Figure 7). Interestingly, the adopted isosteric replacement completely changed the pattern of inhibition of GSTP1-1 catalytic activity; compound **4**, like **1**, was able to fully abolish the enzymatic activity of GSTP1-1, while compound **3** inhibited enzyme activity no more than 50% (Figure 5). Molecular docking orientations obtained for **3** within the GSTP1-1 active site showed, among the clusters with the most favourable binding energies, a pose in which the benzoyl ring of **3** is placed at the interface between the protein monomers, in close proximity with the side chain of Tyr 49^17^. It is possible to speculate that this binding mode could account for the half-site inhibition of GSTP1-1 observed in the presence of **3**. However, the very similar binding pose and energy found also for **4** suggest a more complex enzyme inhibition mechanism that cannot be inferable from the static picture provided by docking. Nevertheless, compound **4** was still capable of affecting formation of the TRAF2-GSTP1-1 complex even in the absence of GSH (Figure 6). These findings suggest that the presence of the ester oxygen atom is not a structural requisite to interfere with the interaction between TRAF2 and GSTP1-1. The highest affinity poses of **3** and **4** show the terminal benzoyl moiety in close contact with the hydrophobic Phe 8 residue. The steric hindrance of the benzoyl moiety in that position can account very well for the restricted mobility of helix 2 of GSTP1-1, which was demonstrated to be crucial for TRAF2 binding^30^. Similar poses are found in the high scoring results of docking on the Apo-GST structures, thus explaining the inhibition of TRAF2-GSTP1-1 binding even in the absence of GSH. The lack of influence of GSH on the ability of compound **4** to disrupt the interaction between TRAF2 and GSTP1-1 is a relevant feature since GSH levels fluctuate in healthy mammalian organs^31^^,^^32^, in several human tumours^3^, as well as in the brain of patients affected by neurodegenerative disorders^15^.

Notably, the presence of a bulky hydrophobic moiety in the side chain of the NBD scaffold of **3** and **4** hinders nucleophilic aromatic substitution reactions. Indeed, like **3**, compound **4** exhibited a stability greater than **1** and **2** when incubated with GSH under physiological conditions (Figure 4). Since ongoing *in vitro* drug metabolism studies indicate that **1** undergoes inactivation by both glucuronidation and oxidation of the side-chain hydroxyl group (Di Paolo et al., unpublished data), replacement of the hydroxyl group of **1** with the benzoyl amide moiety eliminates these possible metabolic inactivation pathways. Finally, the herein reported *in vitro* antitumor activity experiments suggest a correlation between the high stability of **4** and its excellent cytotoxic activity towards human melanoma cells. In light of these results, future studies will be aimed mainly at identifying a suitable formulation to allow the *in vivo* delivery of this novel antitumor NBD derivative.
